# Temporal trends and regional disparities in cancer screening utilization: an observational Swiss claims-based study

**DOI:** 10.1186/s12889-020-10079-8

**Published:** 2021-01-05

**Authors:** Caroline Bähler, Beat Brüngger, Agne Ulyte, Matthias Schwenkglenks, Viktor von Wyl, Holger Dressel, Oliver Gruebner, Wenjia Wei, Eva Blozik

**Affiliations:** 1 Department of Health Sciences, Helsana Group, Zürichstrasse 130, 8600 Dübendorf, Switzerland; 2 Department of Epidemiology, Epidemiology, Biostatistics & Prevention Institute, University of Zurich, Hirschengraben 84, 8001 Zurich, Switzerland; 3 Division of Occupational and Environmental Medicine, Department of Epidemiology, Epidemiology, Biostatistics & Prevention Institute, University of Zurich and University Hospital Zurich, Hirschengraben 84, 8001 Zurich, Switzerland; 4 Department of Geography, University of Zurich, Winterthurerstrasse 190, 8057 Zurich, Switzerland; 5 Institute of Primary Care, University of Zurich and University Hospital Zurich, Pestalozzistrasse 24, 8091 Zurich, Switzerland

**Keywords:** Cancer screening, Mammography, Colonoscopy, Prostate-specific antigen testing, Temporal analysis

## Abstract

**Background:**

We examined colorectal, breast, and prostate cancer screening utilization in eligible populations within three data cross-sections, and identified factors potentially modifying cancer screening utilization in Swiss adults.

**Methods:**

The study is based on health insurance claims data of the Helsana Group. The Helsana Group is one of the largest health insurers in Switzerland, insuring approximately 15% of the entire Swiss population across all regions and age groups. We assessed proportions of the eligible populations receiving colonoscopy/fecal occult blood testing (FOBT), mammography, or prostate-specific antigen (PSA) testing in the years 2014, 2016, and 2018, and calculated average marginal effects of individual, temporal, regional, insurance-, supply-, and system-related variables on testing utilization using logistic regression.

**Results:**

Overall, 8.3% of the eligible population received colonoscopy/FOBT in 2014, 8.9% in 2016, and 9.2% in 2018. In these years, 20.9, 21.2, and 20.4% of the eligible female population received mammography, and 30.5, 31.1, and 31.8% of the eligible male population had PSA testing. Adjusted testing utilization varied little between 2014 and 2018; there was an increasing trend of 0.8% (0.6–1.0%) for colonoscopy/FOBT and of 0.5% (0.2–0.8%) for PSA testing, while mammography use decreased by 1.5% (1.2–1.7%). Generally, testing utilization was higher in French-speaking and Italian-speaking compared to German-speaking region for all screening types. Cantonal programs for breast cancer screening were associated with an increase of 7.1% in mammography utilization. In contrast, a high density of relevant specialist physicians showed null or even negative associations with screening utilization.

**Conclusions:**

Variation in cancer screening utilization was modest over time, but considerable between regions. Regional variation was highest for mammography use where recommendations are debated most controversially, and the implementation of programs differed the most.

**Supplementary Information:**

The online version contains supplementary material available at 10.1186/s12889-020-10079-8.

## Background

In Switzerland, cancer was the second most common cause of death in 2017 [1]. Cancer was shown to have overtaken cardiovascular diseases as the leading cause of death in 12 European Union countries [2]. In Switzerland as well as internationally, public health officials and disease advocacy groups have worked hard in the past years to persuade the population of the importance of targeted cancer screening. These efforts have led to an increased uptake of screening, both in Switzerland and internationally, and have yielded intended results. For example, screening colonoscopy was associated with decreased colorectal cancer incidence and mortality [3, 4]. The proportion of colorectal cancer deaths preventable by colonoscopy use within 10 years has been estimated to be 30.7% in Germany, and 33.9% in the United States [5]. However, while many guidelines consistently recommend the use of some preventive measures, such as colorectal cancer screening in certain age groups [6], other screenings, such as breast cancer screening, continue to be controversial because it is unclear whether lifetime benefits outweigh harms and costs in individuals [7]. Many adults receive routine cancer screening even in old age when it is no longer recommended [8]. The burden associated with overdiagnosis and overtreatment is becoming an increasingly recognized issue.

In Switzerland, colorectal cancer screening is recommended routinely between the age of 50 and 69 years, while routine screening of prostate cancer is discouraged without prior comprehensive education of the patient on benefits and harms and shared decision-making [6, 9]. In fact, prostate-specific antigen (PSA)-based screening without prior informed decision making is one of five listed procedures to be avoided in the ambulatory sector, according to the Swiss Society of Internal Medicine (www.smartermedicine.ch). Breast cancer screening is often recommended, but this recommendation is debated in Switzerland [7, 10]. Since 2011, an increasing number of cantons established breast cancer screening programs. Overall, the implementation of cancer screening programs differs considerably between cantons. Previous research, mainly based on the years 2007 to 2012, has found substantial temporal and regional variation in cancer screening utilization for all three cancer types in Switzerland [11–13]. Thereby, screening rates were generally higher in urban areas and in French- and Italian-speaking regions compared to German-speaking region [11–13]. While breast cancer screening utilization decreased over time in Switzerland, as well as in Europe and the US [13–15], colorectal cancer screening utilization seemed to increase [12, 16]. Prostate cancer screening utilization varied with increasing numbers in Switzerland and Sweden and decreasing trends in the US [11, 17, 18]. However, more recent findings are lacking.

Besides system-related factors, like the existence of national or cantonal screening programs, further factors seem to play a role in whether or not persons participate in cancer screening, such as individual and supply-related variables [8, 19–22]. Furthermore, the patient’s type of health insurance plan and healthcare utilization (such as physician consultations) were associated with cancer screening utilization [23, 24]. Knowledge in the field of cancer screening coverage and its related factors is important for healthcare providers and policymakers as well as for patients when debating on the future directions of planning and resource allocation. Real-world and updated data obtained from routinely collected sources such as health insurance claims are particularly suitable for the study of screening coverage, because they are not collected by means of self-reporting and, as such, results are not distorted due to inherent recall bias [25]. We therefore aimed to examine colorectal, breast, and prostate cancer screening utilization in the appropriate target populations in the years 2014, 2016, and 2018. Moreover, we aimed to identify factors potentially modifying cancer screening utilization in Swiss adults, based on health insurance claims data.

## Materials and methods

### Study design and study population

This is a retrospective, observational study based on insurance claims data of adults, who were insured at Helsana Group in the period from January to December of the years 2014, and/or 2016, and/or 2018, and also in the year preceding each applicable cross-section. The Helsana Group is one of the largest health insurers in Switzerland, insuring approximately 15% of the entire Swiss population across all regions and age groups. Health insurance is mandatory for all Swiss citizens and is based on a cost sharing obligatory basic coverage consisting of deductibles and co-payments. The height of the deductible ranges from Swiss Francs (CHF) 300 to 2500 and can - to some extent - be chosen by the insured person, whereby higher deductibles lead to lower premiums. Co-payments amount to 10% of the yearly healthcare costs and are limited to CHF 700 per person per year. On top of the mandatory insurance, citizens can buy supplementary hospital insurance, which covers further comfort of (semi-)private wards, free choice of physician, and speed of access to elective procedures.

In Switzerland, colorectal cancer screening is recommended routinely between the age of 50 and 69 years, using fecal occult blood testing (FOBT) biennially or colonoscopy every 10 years [6]. These opportunistic screenings are reimbursed by mandatory health insurance since 2013 but are not exempted from deductible. Cantonal screening programs exist since January 1st, 2015 in the canton of Uri, and since September 1st, 2015 in the canton of Vaud. Screenings at the ages 50 to 69 years within these cantonal programs are exempted from deductible, but participants still owe a 10% co-payment. Further cantonal programs did not start before 2019. So, in the years 2016 and 2018, 90.4 and 90.5% of the Swiss population lived in a canton without a colorectal cancer screening program. Between the age of 50 and 69 years (or 74, depending on the canton), mammography is recommended for breast cancer screening biennially [26]. Opportunistic screenings are reimbursed by mandatory health insurance, but they lack quality control of mammography and are not systematically monitored [10]. All mammography screenings in the context of breast cancer screening programs (programmatic screenings) in the cantons of Thurgau, Neuchâtel, Fribourg, Jura, Geneva, Bern, Valais, Vaud (for women between ages 50–74 years), as well as in the cantons of Grisons and St. Gallen (for women between ages 50–69 years) are exempted from deductible, but participants still owe 10% co-payment, except for Jura (up to December 31st 2017) and Valais (up to December 31st 2016), where co-payments are covered by a foundation. In mid-2014, a cantonal screening program was introduced in Basel, and beginning 2015, a further program started in the canton of Ticino (for women aged 50–69 years). Taken together, in the years 2014, 2016, and 2018, 50.6, 40.8 and 41.0% of the Swiss population lived in a canton without a breast cancer screening program. Finally, routine screening of prostate cancer is discouraged by guidelines [9]. No national or cantonal screening program exists.

For each of the observed years, men or women aged 50 to 74 years were considered eligible for prostate or breast cancer screening, respectively, while individuals aged 50 to 69 years were considered eligible for colorectal cancer screening. Collectively across all three data cross-sections, 10.3% individuals of the colorectal, 8.0% of the breast, and 6.3% of the prostate cancer screening populations with missing data (enrollees without full coverage during the observation time, enrollees living abroad, Helsana employees, and enrollees seeking asylum) were excluded. Consequently, the final study population for colorectal cancer screening comprised 270′576, 261′682, and 244′328 individuals in the year 2014, 2016, and 2018, respectively. The corresponding numbers were 171′186, 166′675, and 165′328 for breast, as well as 160′661, 157′269, and 155′944 for prostate cancer screening.

The present study falls outside the scope of the Swiss Federal Act on Research involving Human Beings (Human Research Act, HRA), because it is retrospective and based on anonymized routine administrative claims data. No informed consent from patients or further ethics approval was needed, as all requirements of article 22 of the Swiss data protection law were fulfilled. This was confirmed by a waiver of the ethics committee (Kantonale Ethikkommission Zürich, dated January 11, 2017).

### Measures

Inpatient and outpatient codes used to identify screening services have been published elsewhere [27]. In short, colonoscopy, mammography and PSA testing were used to define colorectal, breast or prostate cancer screening utilization, regardless of whether the tests were used for screening or diagnostic purposes. In contrast to Ulyte et al., we additionally considered FOBT as a colorectal cancer screening test. Sociodemographic factors (sex and age), health-related factors (number of chronic conditions assessed by means of the Pharmacy-based Cost Group (PCG) model [28], and having had a major surgery or disease associated with the specific cancer of interest, based on inpatient and outpatient diagnoses and treatments in the preceding year (specific disease)), as well as the patient’s type of health insurance plan (supplementary hospital insurance, managed care, and deductible level) were included as explanatory variables. Regional (urban/ rural residence and language region (German, French or Italian)) and system-related factors (existence of a cantonal screening program) were also considered. In the present data set, adults from two cantons belonging to two different language regions were enrolled; the canton of Bern (BE) incorporates German-speaking and French-speaking regions, and the canton of Grisons (GR) incorporates German-speaking and Italian-speaking regions. The Rhaeto-Romanic region of GR (hosting < 1% of inhabitants) was assigned to the German-speaking region. Furthermore, screening specific specialist physician density information of the corresponding year was provided by the Swiss Medical Association (FMH) and included as supply-related factor (gastroenterologist in colonoscopy/FOBT, gynecologist in mammography, and urologist in PSA testing utilization). Finally, beyond the respective screening (specific testing), the following healthcare utilization measures were considered as explanatory variables: the number of physician consultations, total healthcare costs, and at least one acute hospital admission, all measured in the preceding year, as well as colonoscopy/FOBT in the same year (for mammography and PSA testing analysis).

Most variables were originally measured on a nominal scale. All continuously measured variables were transformed into categories before their use in regression analysis (age (five-year groups), height of deductible (above CHF 500 yes/no), specialist physician density (above median density yes/no), number of chronic conditions (none, one, multiple), number of physician consultations (quarters), healthcare costs (quarters), and acute hospital admissions (at least one yes/no).

### Statistical analysis

The baseline characteristics of all included study subjects are presented as counts and percentages, or as mean and standard deviation for continuous variables. For each of the three screening types, we compared subjects with and without the respective testing. We calculated the testing prevalence per year (2014, 2016, 2018) for each cancer screening type, and we then tested whether the testing prevalences were equal using Chi-squared tests, pairwise between years (with Holm correction for multiple testing), as well as across all 3 years. Additionally, we calculated the age-standardized testing prevalence per canton. Small cantons with low numbers of observations were grouped with a neighboring canton where sensible (Appenzell Innerrhoden and Appenzell Ausserrhoden, Neuchâtel and Jura, Obwalden and Nidwalden for colorectal cancer screening, and Uri and Glarus for breast and prostate cancer screening). Furthermore, a simple probability-rate-probability conversion (assuming constant testing rates) was performed to estimate the longer-term testing prevalence, thereby taking the recommended screening interval into account [29].

In logistic regression models with testing in a given year as outcome variable, we calculated the average marginal effect, i.e. the averaged difference in the predicted probability of having the outcome, for each of the included covariates [30, 31]. In conjunction with the fact that all included covariates are categorical, the average marginal effects facilitate the interpretation of each association (direction and magnitude) between each covariate and the outcome on the probability scale. This exploratory analysis was performed on the pooled data of all three cross-sections, for each screening type separately. One assumption in logistic regression is the independence of all observations, which is violated in the pooled cross-sections where some subjects are observed in more than one cross-section. This violation could have led to a wrong estimation of the variance in the effect estimates. A sensitivity analysis using clustered covariance matrix estimation with individuals as clusters showed similar interval estimates for most covariates [32–34]. Since we have very few (one to three) observations per cluster, these estimations may not work well, and we therefore show these results as supplementary material only (Additional file 3) [34].

All analyses were performed using R version 3.6.1.

## Results

Overall, the mean (sd) age was 59.1 (5.95) years, 61.7 (7.37) years, and 61.6 (7.58) years, respectively, in the colorectal, breast and prostate cancer screening populations. Men were slightly under-represented in the colorectal cancer screening population (48.3%). Characteristics of the populations for the year 2018 are shown in Table 1, and corresponding characteristics for the years 2014 and 2016 are available as supplementary material (Additional files 1 and 2).

**Table 1:** Characteristics of eligible population receiving colonoscopy/FOBT, mammography or PSA testing in 2018

	_Colonoscopy/FOBT_^a^	_No Colonoscopy/FOBT_^a^	Mammo-graphy	No Mammography	_PSA testing_^b^	_No PSA testing_^b^
N (%)	22′453 (9.2)	221′875 (90.8)	33′747 (20.4)	131′581 (79.6)	49′521 (31.8)	106′423 (68.2)
Male sex (%)	10′876 (48.4)	107′027 (48.2)	0 (0)	0 (0)	49′521 (100)	106′423 (100)
Age in years (mean, sd)	59.8 (5.94)	59.0 (5.90)	61.1 (7.17)	61.9 (7.43)	64.0 (7.36)	60.5 (7.48)
High deductible (%)	4′869 (21.7)	67′033 (30.2)	5′990 (17.7)	30′831 (23.4)	10′204 (20.6)	38′822 (36.5)
Managed care (%)	13′708 (61.1)	134′626 (60.7)	20′684 (61.3)	78′601 (59.7)	29′346 (59.3)	63′850 (60.0)
Suppl. hospital insurance (%)	5′620 (25.0)	48′174 (21.7)	9′795 (29.0)	33′285 (25.3)	12′909 (26.1)	20′838 (19.6)
Language region						
German (%)	16′659 (74.2)	172′012 (77.5)	19′994 (59.2)	106′389 (80.9)	35′531 (71.7)	85′961 (80.8)
French (%)	3′629 (16.2)	34′750 (15.7)	9′297 (27.5)	17′196 (13.1)	8′724 (17.6)	14′485 (13.6)
Italian (%)	2′165 (9.6)	15′113 (6.8)	4′456 (13.2)	7′996 (6.1)	5′266 (10.6)	5′977 (5.6)
Urban region (%)	17′527 (78.1)	169′001 (76.2)	26′611 (78.9)	101′054 (76.8)	38′503 (77.8)	79′373 (74.6)
Major related surgery/ disease (%)	269 (1.2)	696 (0.3)	2′022 (6.0)	1′164 (0.9)	2′398 (4.8)	1′011 (0.9)
Chronic conditions (mean, sd)	1.8 (1.87)	1.4 (1.70)	1.8 (1.87)	1.6 (1.85)	2.0 (1.79)	1.3 (1.66)
Cantonal program (%)	1′359 (6.1)	12′526 (5.6)	21′654 (64.2)	61′795 (47.0)	0 (0)	0 (0)

^a^ *FOBT* Fecal occult blood testing

^b^ *PSA* Prostate-specific antigen

Overall, 8.3% of the eligible population received colonoscopy/FOBT in 2014, 8.9% in 2016, and 9.2% in 2018. This corresponds to a small, but statistically significant increase between 2014 and 2018 (Table 2). While the proportion of persons with FOBT decreased (from 2.5% in 2014 to 2.1% in 2018), the number of persons with colonoscopy slightly increased (from 6.1% in 2014 to 7.4% in 2018). Regarding breast cancer screening, 20.9, 21.2, and 20.4% of the eligible population had mammography in 2014, 2016, and 2018, respectively. The corresponding numbers of PSA testing in the eligible prostate cancer screening population were 30.5, 31.1, and 31.8%. Assuming constant utilization rates, approximately 58% of eligible persons were estimated to have had colorectal cancer screening (54% with colonoscopy within 10 years, and 4% with FOBT within 2 years), and about 37% to have had breast cancer screening (mammography within 2 years) by 2018.

**Table 2:** Proportions of the eligible populations with colonoscopy/FOBT, mammography or PSA testing in 2014, 2016, and 2018, and significance tests of change of these proportions, pairwise between years

	2014	2016	2018	Change in proportion 20,142,016	*p*^*a*^	Change in proportion 2016–2018	*p*^*a*^	Change in proportion 2014–2018	*p*^*a*^
colonoscopy/ FOBT^b^	8.3%	8.9%	9.2%	0.7%	< 0.001	0.2%	0.003	0.9%	< 0.001
mammography	20.9%	21.2%	20.4%	0.3%	0.017	−0.8%	< 0.001	−0.5%	< 0.001
PSA testing^c^	30.5%	31.1%	31.8%	0.5%	0.001	0.7%	< 0.001	1.2%	< 0.001

^a^ using Chi-squared tests

^b^ *FOBT* Fecal occult blood testing

^c^ *PSA* Prostate-specific antigen

**Fig. 1:**
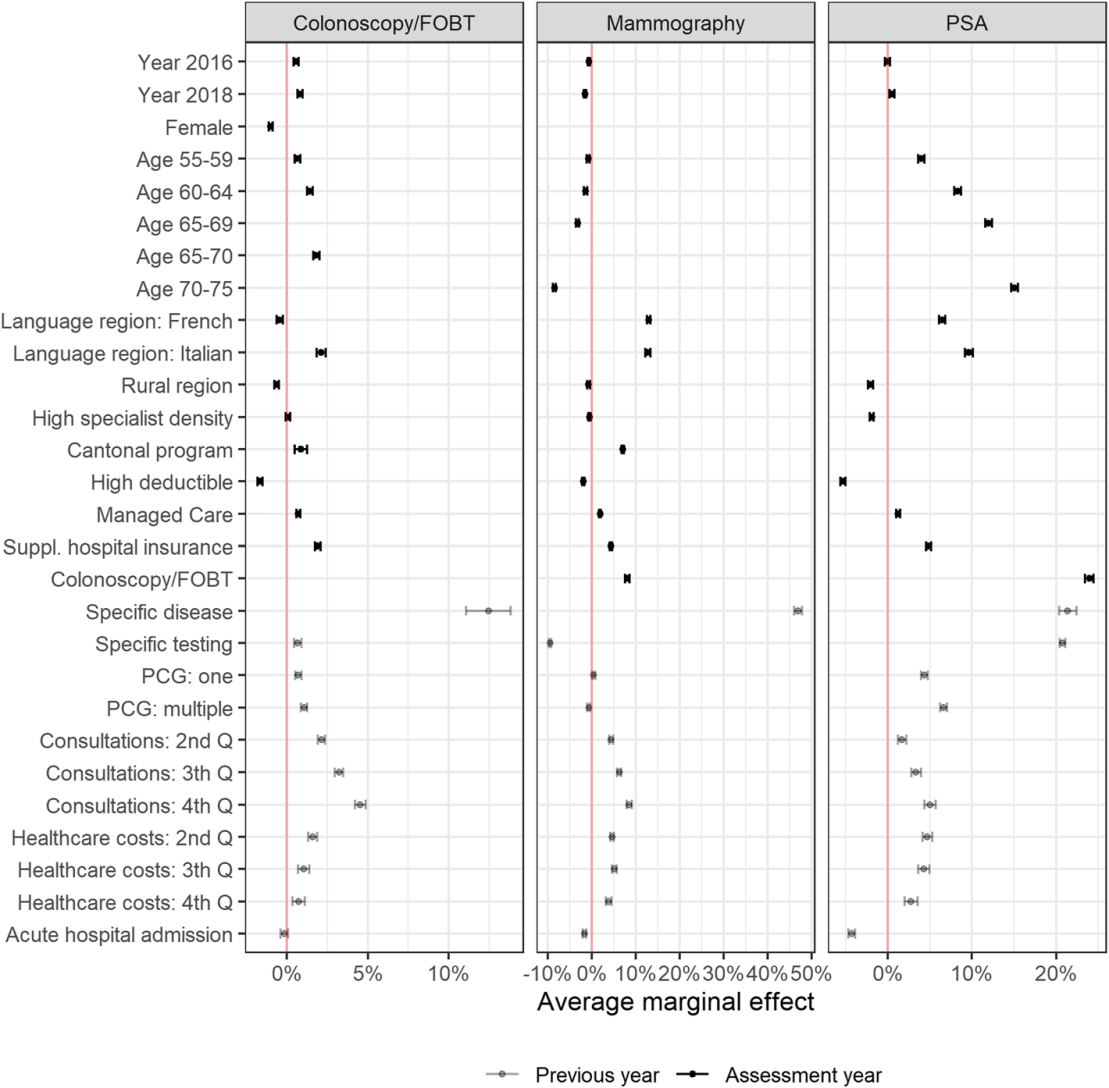
Standard estimates of the average marginal effects on colonoscopy/FOBT, mammography and PSA testing utilization. FOBT = fecal occult blood testing; PCG = Pharmacy-based Cost Group

Looking at the adjusted testing utilization in multivariable regression analysis, there was a slight increase in colonoscopy/FOBT utilization of 0.6% (CI: 0.4–0.7%) in 2016 and 0.8% (CI: 0.6–1.0%) in 2018 as compared to 2014 (Fig. 1). These adjusted increases correspond to the raw increases of 0.6% between 2014 and 2016, and of 0.8% between 2014 and 2018 (Table 2). Mammography utilization slightly decreased by 0.6% (CI: 0.3–0.8%) in 2016 and by 1.5% (CI: 1.2–1.7%) in 2018, compared to 2014. This is contradictory to the minimal increase of 0.3% between 2014 and 2016, but similar to the slight decrease of 0.5% between 2014 and 2018, when looking at the raw proportions. The utilization of PSA testing remained unchanged in 2016 and increased slightly in 2018 (by 0.5%; CI 0.2–0.8%) compared to 2014. These slightly attenuated results are comparable to the changes in raw proportions.

In multivariable regression analysis, several determinants were associated with testing utilization (Fig. 1 and Additional file 3). Utilization increased with increasing age for colonoscopy/FOBT and even more strongly for PSA testing but decreased slightly with increasing age for mammography use. Being female was associated with a 1% (CI: 0.9–1.1%) lower probability of receiving colonoscopy/FOBT. Having had a major surgery or disease associated with the specific cancer of interest was strongly related to receiving colonoscopy/FOBT, mammography, or PSA testing in the observed year, although this applied to a small proportion of patients. Having one or more chronic conditions was positively associated with colonoscopy/FOBT and PSA testing, whereas multiple chronic conditions were slightly negatively associated with mammography use. Regarding the patient’s type of health insurance plan, having supplementary hospital insurance was consistently associated with a 1.9 to 4.8% higher probability of testing utilization, depending on cancer type, while having a higher deductible was consistently associated with a 1.7 to 5.3% lower probability of testing utilization. The positive effect of being in a managed care model on testing utilization was minimal, but slightly higher for mammography use than for receiving colonoscopy/FOBT.

When compared to the German-speaking region, living in the Italian-speaking region was associated with a higher probability of receiving colonoscopy/FOBT, whereas living in the French-speaking region had almost no effect. In contrast, living in the French- and the Italian- compared to the German-speaking region increased mammography use by 13.0% (CI: 12.6–13.4%) and 12.8% (CI: 12.3–13.3%), respectively, and PSA testing by 6.5% (CI: 6.1–6.8%) and 9.6% (CI: 9.2–10.1%), respectively. The average marginal effect of living in the rural area on testing utilization was negative, but mostly small, for all cancer types. The existence of a cantonal program had a positive impact of 7.1% (CI: 6.8–7.4%) on mammography utilization, as well as a small positive impact of 0.9% (CI: 0.5–1.2%) on colonoscopy/FOBT utilization. While the cantonal density of gastroenterologists and gynecologists seemed to have no influence on colonoscopy/FOBT and mammography utilization, the cantonal density of urologists was negatively associated with PSA testing.

High healthcare utilization, assessed by higher healthcare costs and more physician consultations in the preceding year, were both associated with a higher probability of being tested for all cancer types. Furthermore, receiving colonoscopy/FOBT in the corresponding year was highly related to mammography use and PSA testing in the observed year. In contrast, the respective testing in the previous year showed varying associations: Colonoscopy/FOBT in the previous year was associated with a slightly higher probability of receiving colonoscopy/FOBT in the observed year. Previous PSA testing was strongly associated with re-testing in the observed year, while mammography in the previous year was negatively associated with present mammography utilization. All the above-mentioned observed effects hardly changed when different observations periods were analyzed separately (results not shown).

**Fig. 2:**
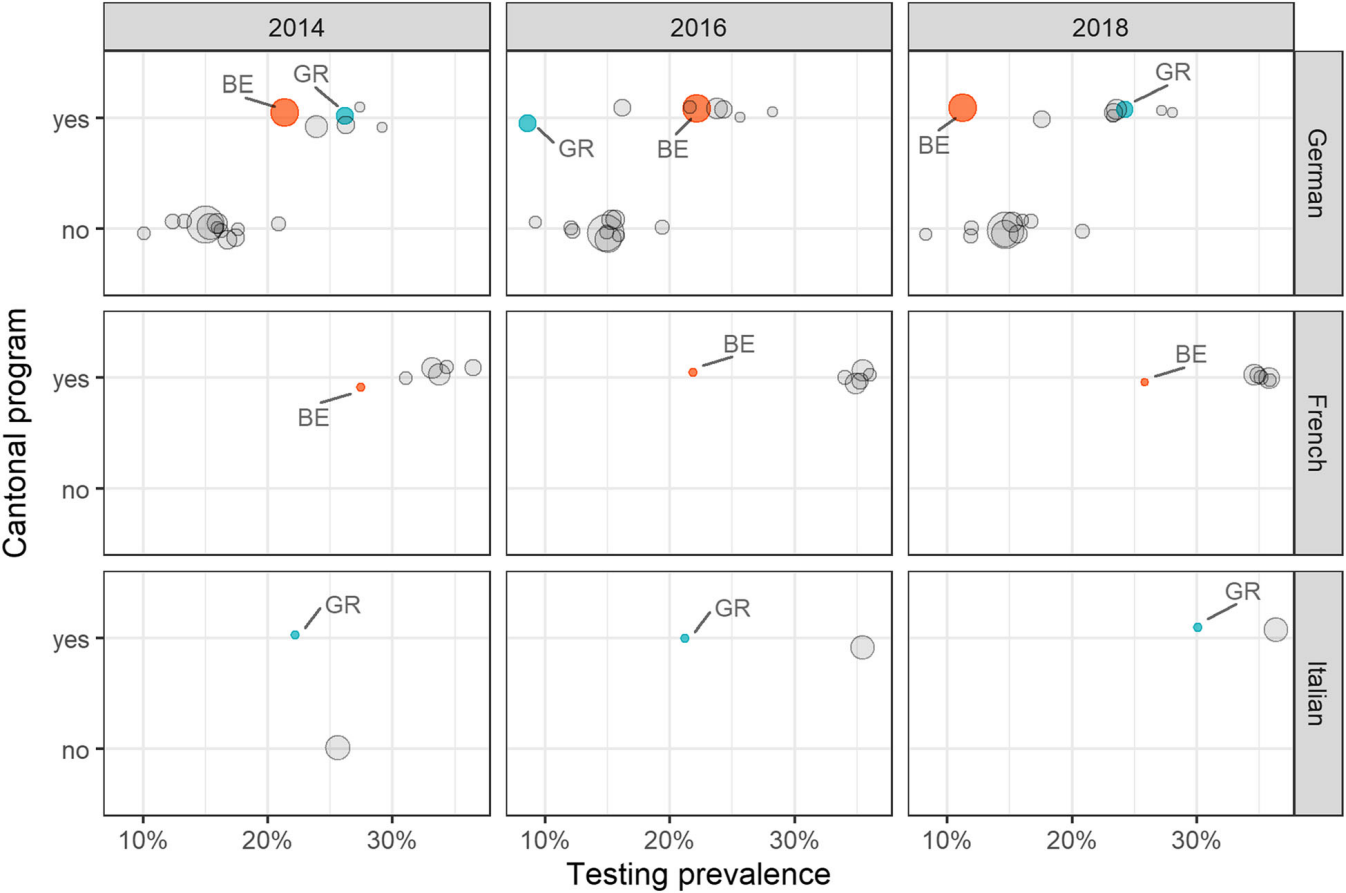
Age-standardized proportions of mammography utilization per canton, by language region and cantonal screening program. BE = canton of Bern; GR = canton of Grisons. The columns illustrate different temporal cross-sections and the rows show different dominant languages in cantons. Cantons in each row are further stratified by the existence of cantonal programs. BE and GR, which are bilingual cantons, are shown in both linguistic rows. The size of the bubbles corresponds to the population size of the canton

Looking at the regional distribution of age-standardized testing utilization, we found significant differences between the three language regions on the one hand, and between cantons with and without screening programs on the other hand. Since these interactions cannot be captured by adjusted regression modelling, we illustrate this interrelation in Fig. 2.

In bilingual cantons incorporating more than one language region (BE, GR), both, the existence of a program and the language region seem to influence screening participation. In the German-speaking regions, the age-standardized mammography utilization rates were approximately 7% higher in cantons with a breast cancer screening program compared to cantons without such a program. Noteworthy, in 2018 in the German-speaking part of Bern, where screening programs were reorganized in 2017/2018, the utilization rate was significantly lower than in the French-speaking part of Bern, where a screening program was jointly established together with the cantons of Jura and Neuchâtel in 2011. As all French-speaking regions belonged to cantons with existing screening programs, the effect of a program in these regions could not be evaluated. In the Italian-speaking region (mainly represented by the canton of Ticino), the utilization rate increased significantly after the introduction of the cantonal screening program in 2015. In contrast, this increase was much smaller in the canton of Grisons, where only a small part of the population lives in an Italian-speaking region. The supplement shows corresponding bubble plots for colonoscopy/FOBT use and PSA testing (Additional files 4 and 5).

## Discussion

Variations in cancer screening utilization were modest over time, but considerable between regions. Regional variation was highest for mammography use where recommendations are debated most intensively, and the implementation of programs differed considerably. The present study showed an increasing trend of 0.8% (0.6–1.0%) for colonoscopy/FOBT and of 0.5% (0.2–0.8%) for PSA testing, while mammography decreased by 1.5% (1.2–1.7%) between 2014 and 2018.

Although colorectal cancer screening by means of colonoscopy or FOBT is clearly recommended and has been promoted since 2014 in Switzerland, e.g. by pharmacies, colonoscopy/FOBT utilization in this population-based study was rather low and has hardly changed since then. Considering the recommended ten-year screening interval for colonoscopy and the two-year interval for FOBT, about 58% of the eligible population would have been tested by 2018. This proportion is slightly higher compared to previous Swiss and Italian findings, but slightly lower to screening participation in the US. In a Swiss cross-sectional study conducted in 2017 [35], 41% of patients who visited a primary care physician had a colonoscopy within 10 years and 4% had a FOBT within 2 years. According to an earlier population-based Swiss survey in 50 to 75 year old persons, colorectal cancer screening defined as endoscopy (either colonoscopy or sigmoidoscopy) in the past 10 years or FOBT in the past 2 years increased from 18.9% in 2007 to 22.2% in 2012; this increase within 5 years was more substantial compared to what we found, and was due to growing endoscopy numbers in 2012, while FOBT decreased [12]. The overall higher screening utilization in our study might be owed to the addition of colorectal cancer screening to the benefit basket of the basic insurance coverage in Switzerland in 2013. Moreover, our inability to discriminate between diagnostic and screening colonoscopy/FOBT, and the differences in study designs (claims-based versus survey-based) might partially explain the different findings. A recent Italian study in women aged 50–54 years found participation rates within the last 2 years for colorectal cancer screening (FOBT) of 45.1% [36]. In the US, 64.5% of respondents aged 50 to 75 years reported having participated in colorectal cancer screening by 2010: [37].

The slight decline in mammography utilization in our study was similar to previous Swiss and European findings. For example, the proportion of Swiss women with any mammography in the last 12 months decreased from 19.1% in 2007 to 11.7% in 2012 in a survey data-based study [13]. Annual participation rates for breast cancer screening varied between 23 and 84% in 17 European countries with mostly organized national or regional breast screening programs, with a decreasing trend even before 2014 [14]. Thus, mammography use within 1 year of approximately 20% (or 37% within 2 years) presented in our study is low when compared internationally. In a recent Italian study, mammography use within 2 years amounted to 85.1% [36]. Moreover, mammography use increased from 48% in 2007/08 to 54% in 2011/12 in a German city after the implementation of a mammography screening program by the end of 2005 [38]. The decline found in our analysis is likely to be influenced by the public debate about benefits and harms of breast cancer screening [39]. By the end of 2013, the Swiss Medical Board recommended that no new systematic mammography screening programs be introduced in Switzerland due to lack of cost-effectiveness and undesirable effects outweighing desirable effects [40, 41]. The relative risk reduction or lifesaving effect is small, while false-positive results and overdiagnosis can cause considerable harm in screened patients [42, 43]. Therefore, a more personalized approach is now recommended in the US, meaning that physicians should have a more informed discussion with patients.

Our analysis showed that proportions of PSA testing remained above 30% between 2014 and 2018. These rates seem rather high, given the uncertainty of the usefulness of PSA screening and the potential harm caused by overdiagnosis and by associated overtreatment. This is the reason why most organizations and Societies in Europe and America, as well as the Swiss Medical Board, recommend against routine PSA screening without prior shared decision making [9, 44]. According to our findings, the impact of the top five list by the Smarter Medicine Initiatives (www.smartermedicine.ch), published in 2014, does not seem to have considerably impacted PSA testing rates. Our results are in line with former Swiss findings. Between 1992 and 2012, use of PSA screening within the last 2 years increased from 32.6 to 42.4% in Swiss men aged 50 years and older [11]. In contrast, a US study demonstrated a decline in PSA testing after the publication of the 2012 USPSTF recommendation discouraging testing in asymptomatic men [44]. Since PSA testing in our study is strongly associated with further measures of healthcare utilization like the number of consultations in the preceding year and concurrent colonoscopy/FOBT use, and is mainly related to patients with low deductibles and with multiple chronic conditions, we might speculate that this specific testing is done additionally in the course of other medical examinations as no special equipment is needed.

In general, recommended screenings like colorectal cancer screening have not clearly increased and discouraged screenings like prostate cancer screening have not clearly decreased over time. However, the present results need to be interpreted with caution, as we were unable to discriminate between screening and diagnostic or follow-up testing (except if screening occurred within a cantonal program and was reimbursed as such). Particularly in patients with a major related surgery or disease, the colonoscopy/FOBT, mammography or PSA testing might be attributable to diagnostic or follow-up testing rather than screening purposes. This holds especially true for colonoscopy which is only recommended once in 10 years. However, the number of patients with related disease or surgery is low.

Screening utilization was associated with a variety of individual, regional, insurance-related, as well as with supply-, and system-related factors. The direction of the average marginal effects on testing utilization are comparable across all cancer types for most of these factors. However, age was positively associated with colonoscopy/FOBT and PSA testing, but inversely associated with mammography. The decline in the latter in older age is mostly owed to a lower probability in women aged over 70 years where screening is no longer supported by all cantonal programs. Being male was associated with a higher prevalence of colonoscopy/FOBT use, similar to a former Swiss study conducted in 2012 [12], but contrary to a Flemish study, where utilization rates in 2013 and 2014 were lower for men [45].

Having supplementary hospital insurance was consistently associated with a higher, while having a higher deductible with a lower probability of screening utilization. Similar findings were found for colorectal [46], as well as for breast cancer screening [13, 47]. The marginal positive effect of being in a managed care model on cancer screening utilization is in line with a previous study showing positive associations, where slightly higher effects were observed for breast than for colorectal cancer screening as well [24]. Though, the effects observed in our study are small.

Screening utilization was generally more likely in the French- and Italian-speaking regions compared to the German-speaking region, except for colonoscopy/FOBT use, where living in the French-speaking region hardly had any effect. Regional variation was highest for mammography use, where recommendations are debated most and the implementation of programs differed considerably. Correspondingly to our findings, significant differences in breast cancer screening attendance between women in the French- and the German-speaking region were found in the study by Eichholzer et al. [48] and Fenner et al. [13] Alike, prostate cancer screening rates were higher in men living in the French- or Italian- as compared to the German-speaking region, and in urban rather than rural areas [11]. The proportions of patients with either FOBT or colonoscopy also varied widely between language regions [35] The increased number of screening programs as well as the higher screening utilization even in the absence of specific programs might point to a diverse attitude of patients and/or physicians towards preventive measures in the French- and Italian- compared to the German-speaking region.

Cantonal programs for breast and colorectal cancer screening were associated with a small, but significant increase in testing utilization, although the association was stronger in the former, since only two cantonal colorectal cancer screening programs were in place by 2018, and because the overall proportion of persons receiving colonoscopy/FOBT was rather low. Generally, despite an increasing number of cantons offering breast cancer screening programs since 2011, the overall marginal effect showed a decreasing trend in mammography utilization. This decline is mainly based on cantons without any screening program. Similarly, the decline in mammography screening was more pronounced in cantons with no or with a long-standing screening program in the previous Swiss survey-based study [13]. In contrast, according to another Swiss study looking at data from the Swiss Health Survey in the years 1997, 2002, 2007, and 2012, only a small part of the (relatively high) mammography utilization rates could be attributed to organized programs, and non-use of mammography was not attributable to a lack of information or to financial barriers [47]. Another Swiss study compared participants of opportunistic with participants of organized mammography screening and found that mammography screening programs mainly attracted women in lower socio-economic strata [49]. Unfortunately, we were unable to differentiate between those two screening types by means of our data.

A high density of related specialist physicians had null or even a negative association with screening utilization in our study. This is in contrast to a German online survey where PSA testing was judged as useful by all urologists but only by half of the general practitioners, and where PSA testing practices varied between both clinician groups [50]. Higher PSA screening rates were also seen in regions where the primary care specialist was unlikely to be the predominant physician for ambulatory visits [22]. We can only speculate that PSA testing is done by primary care physicians to a very substantial extent. At least, PSA testing was higher in those with primary care physician visits in the preceding year [11].

High healthcare utilization, assessed by higher healthcare costs and more physician consultations in the preceding year, were both associated with a higher probability of being tested for all three cancer types, although this association was less strong in the highest cost category. In line with our findings, having consulted a primary care physician or a specialist physician in the last 12 months was significantly associated with a higher prevalence of colorectal cancer screening in Switzerland in 2012 [12]. This implies that physicians assume their obligation to talk with their patients about preventive measures like cancer screening. Concurrent colonoscopy/FOBT use increased the probability of mammography or PSA testing. Likewise, US women who adhered to breast cancer screening recommendations were four times more likely to have had colorectal cancer screening [23]. PSA testing in the preceding year also increased PSA testing in the observed year. This finding is congruent to the clinical practice that individuals who are being screened, are screened on a yearly basis. In contrast, mammography in the preceding year was related to lower mammography use in the observed year. This might be indicative of the biennially screening recommendations. Then again, colonoscopy/FOBT in the previous year hardly had any influence. As discussed previously, the inability to discriminate between diagnostic and screening testing on the one hand, and the difference in recommended screening intervals for colonoscopy and FOBT on the other hand, might have influenced these findings.

### Strengths and limitations

Our study has several strengths and limitations worth mentioning. The major strength is the highly reliable and comprehensive, population-based data set available for analysis over three cross-sections. The major limitation is that we were unable to discriminate between screening and diagnostic testing (except if screening occurred within a cantonal program and was reimbursed as such). This misclassification issue leads to an overestimation of screening utilization, which might be more pronounced in breast and prostate cancer screening where comparably more patients had underlying diseases. In contrast, tests that have been paid out-of-pocket were not captured by means of claims data, which is more likely for PSA testing than for colonoscopy or mammography. This may have led to an underestimation of screening utilization. Furthermore, we might have missed some codes used by specific laboratories to account for cancer screening. Second, observations were not necessarily independent between the different observation periods, which may have led to an underestimation of the variance in the effect estimates. However, in a sensitivity analysis using clustered covariance matrix estimation with individuals as clusters, interval estimates altered only marginally (Additional file 3). Third, further aspects influencing cancer screening participation in individuals, like difference in life expectancy [51], screening habits or patient’s preferences [23], could not be taken into account by means of our claims data. Yet, we considered concurrent colonoscopy/FOBT use in the breast and prostate cancer screening population as a proxy for screening habit. Fourth, categorization of continuous variables is sometimes discouraged, because it leads to information loss and assumes a flat relationship between the covariate and the outcome within intervals, which is less likely than e.g. a linear relation in most cases [52]. While these reservations are certainly true, we chose to categorize continuous variables because in the case of this exploratory analysis the loss in precision is outweighed by the increased interpretability of the results.

### Implications

Clinical practice guidelines are an essential step forward to improve patient care and provide recommendations based on a systematic review of evidence [53]. However, although clearly recommended, colorectal cancer screening is still not performed by almost half of the eligible population. Therefore, our findings highlight the need for enhanced awareness of systematic colorectal cancer screening benefits to reduce cancer-specific mortality rates. During a physician consultation or hospitalization, strategies could be employed to counsel, educate, and motivate patients towards preventive measures like cancer screening, particularly for those who are at higher risk of disease. Furthermore, information campaigns and further actions like invitation letters should more specifically address the population who is less likely to be screened, e.g., individuals with a high deductible. Offering of prevention and health promotion to enrollees with supplementary health insurance seem to go in that direction. Additionally, further cantonal programs were established in 2019 to hopefully promote colorectal cancer screening. Yet, a standardization of screening programs and their payments in Switzerland is urgently warranted [54], and might help to increase equal access and uptake.

Unnecessary screening may not only cause adverse effects but also generate high healthcare costs [55]. Regarding prostate cancer screening, annual PSA testing may result in an overdiagnosis rate of 50% [56]. Increased awareness of initiatives such as the Smarter Medicine recommendations of the Swiss Society of Internal Medicine are therefore crucial. It should be noted that screening attendance was shown to be mainly influenced by social norms and role models [57], and not solely by guidelines, even among physicians [53]. Thus, physician training regarding informed decision making as well as the development of improved information and decision aids is warranted [11].

Although breast cancer screening is recommended biennially, and various screening programs exist in Switzerland, mammography use is low. Controversies about the value of screening and further disparities, like regional and system-related differences regarding program implementation, might contribute to these findings. For example, the risk of overdiagnosis and overtreatment has been repeatedly demonstrated and debated, particularly after breast cancer screening implementation [10, 43, 58, 59]. Further promoting interventions for breast cancer screening, as mentioned in the systematic review by Agide et al. [60], may therefore have difficulties in being introduced in Switzerland. However, unless screening participation reaches an acceptable standard level [14], it may not achieve the warranted gains like a reduction in cancer-specific mortality.

## Conclusions

Variations in cancer screening utilization were modest over time, but considerable between regions. Regional variations were highest for mammography use where recommendations are debated most controversially. Since recommended screening (like colorectal cancer screening) has not clearly increased and discouraged screening (like prostate cancer screening) has not clearly decreased over time, health policy adoptions are needed to optimize preventive care in Switzerland.

## Supplementary Information

**Additional file 1.** Characteristics of eligible population receiving colonoscopy/FOBT, mammography or PSA testing in 2014.

**Additional file 2.** Characteristics of eligible population receiving colonoscopy/FOBT, mammography or PSA testing in 2016.

**Additional file 3.** Estimates with clustered covariance of the average marginal effects on colonoscopy/FOBT, mammography and PSA testing utilization.

**Additional file 4.** Age-standardized proportions of colonoscopy/FOBT utilization per canton, by language region and cantonal screening program. The size of the bubbles corresponds to the population size of the canton; BE = canton of Bern; GR = canton of Grisons. The age-standardized proportion of persons with colonoscopy/FOBT mainly differed between the German- and the Italian-speaking regions. By the end of 2015, only two cantons offered a specific program, of which one canton (Uri) is minor. In the canton of Vaud, the colonoscopy/FOBT utilization increased between 2016 and 2018.

**Additional file 5.** Age-standardized proportions of PSA testing utilization per canton, divided by language region. The size of the bubbles corresponds to the population size of the canton; BE = canton of Bern; GR = canton of Grisons. Regarding prostate cancer screening, where no cantonal programs exist, PSA testing utilization seems higher in the French- and Italian-speaking compared to the German-speaking regions. Then again, the testing proportions in the French-speaking regions of BE and the Italian-speaking region of GR are more comparable to the German-speaking region of the respective canton.

## Data Availability

The data that support the findings of this study are available from Helsana (https://www.helsana.ch/en/helsana-group), but restrictions apply to the availability of these data, which were used under license for the current study, and so are not publicly available. Data are however available from the authors upon reasonable request and with permission of Helsana (gesundheitskompetenz@helsana.ch).

## Abbreviations

BE: Canton of Bern
CHF: Swiss Francs
CI: Confidence Interval
FOBT: Fecal occult blood testing
GR: Canton of Grisons
PSA: Prostate-specific antigen
FMH: Swiss Medical Association
USPSTF: US Preventive Services Task Force
