# Supplementary material for: Temporal trends and regional disparities in cancer screening utilization: an observational Swiss claims-based study
Source: BMC Public Health. 2021 Jan 5;21:23. doi: 10.1186/s12889-020-10079-8 (PMC7786957; doi:10.1186/s12889-020-10079-8)
Supplement: Supplementary file 1 — Additional file 1. Characteristics of eligible population receiving colonoscopy/FOBT, mammography or PSA testing in 2014. [file 12889_2020_10079_MOESM1_ESM.docx]

### Additional file 1 Characteristics of eligible population receiving colonoscopy/FOBT, mammography or PSA testing in 2014.

|  | Colonoscopy/FOBT^a^ | No  Colonoscopy/FOBT^a^ | Mammography | No  Mammography | PSA testing^b^ | No  PSA testing^b^ |
| --- | --- | --- | --- | --- | --- | --- |
| N (%) | 22'434 (8.3) | 248'142 (91.7) | 35'775 (20.9) | 135'411 (79.1) | 49'063 (30.5) | 111'598 (69.5) |
| Male sex (%) | 10'974 (48.9) | 119'776 (48.3) | 0 (0) | 0 (0) | 49'063 (100) | 111'598 (100) |
| Age in years  (mean, sd) | 60.3 (5.90) | 59.1 (5.98) | 60.9 (7.10) | 61.8 (7.39) | 64.0 (7.15) | 60.4 (7.44) |
| High deductible (%) | 4'327 (19.3) | 69'127 (27.9) | 5'888 (16.5) | 29'831 (22.0) | 9'125 (18.6) | 37'706 (33.8) |
| Managed care (%) | 11'242 (50.1) | 122'799 (49.5) | 17'917 (50.1) | 65'698 (48.5) | 23'944 (48.8) | 54'694 (49.0) |
| Suppl. hospital insurance (%) | 6'134 (27.3) | 55'484 (22.4) | 10'393 (29.1) | 35'057 (25.9) | 13'123 (26.7) | 21'712 (19.5) |
| Language region |  |  |  |  |  |  |
| German (%) | 17'371 (77.4) | 192'966 (77.8) | 23'554 (65.8) | 107'986 (79.7) | 35'444 (72.2) | 90'146 (80.8) |
| French (%) | 3'020 (13.5) | 37'298 (15.0) | 8'837 (24.7) | 17'396 (12.8) | 8'488 (17.3) | 14'527 (13.0) |
| Italian (%) | 2'043 (9.1) | 17'878 (7.2) | 3'384 (9.5) | 10'029 (7.4) | 5'131 (10.5) | 6'925 (6.2) |
| Urban region (%) | 17'691 (78.9) | 188'959 (76.1) | 27'846 (77.8) | 104'823 (77.4) | 38'179 (77.8) | 83'488 (74.8) |
| Major related surgery/ disease (%) | 273 (1.2) | 799 (0.3) | 2'181 (6.1) | 1'188 (0.9) | 2'199 (4.5) | 941 (0.8) |
| Chronic conditions  (mean, sd) | 1.8 (1.81) | 1.4 (1.67) | 1.8 (1.85) | 1.6 (1.81) | 2.0 (1.75) | 1.3 (1.62) |
| Cantonal program (%) | 0 (0) | 0 (0) | 18'343 (51.3) | 48'431 (35.8) | 0 (0) | 0 (0) |

^a^ FOBT = fecal occult blood testing

^b^ PSA = prostate-specific antigen
