# Supplementary material for: Temporal trends and regional disparities in cancer screening utilization: an observational Swiss claims-based study
Source: BMC Public Health. 2021 Jan 5;21:23. doi: 10.1186/s12889-020-10079-8 (PMC7786957; doi:10.1186/s12889-020-10079-8)
Supplement: Supplementary file 2 — Additional file 2. Characteristics of eligible population receiving colonoscopy/FOBT, mammography or PSA testing in 2016. [file 12889_2020_10079_MOESM2_ESM.docx]

### Additional file 2 Characteristics of eligible population receiving colonoscopy/FOBT, mammography or PSA testing in 2016.

|  | Colonoscopy/FOBT^a^ | No  Colonoscopy/FOBT^a^ | Mammography | No  Mammography | PSA testing^b^ | No  PSA testing^b^ |
| --- | --- | --- | --- | --- | --- | --- |
| N (%) | 23'412 (8.9) | 238'270 (91.1) | 35'388 (21.2) | 131'287 (78.8) | 48'859 (31.1) | 108'410 (68.9) |
| Male sex (%) | 11'362 (48.5) | 115'090 (48.3) | 0 (0) | 0 (0) | 48'859 (100) | 108'410 (100) |
| Age in years  (mean, sd) | 60.0 (5.93) | 59.0 (5.96) | 61.0 (7.14) | 61.9 (7.43) | 64.0 (7.28) | 60.5 (7.47) |
| High deductible (%) | 4'737 (20.2) | 67'993 (28.5) | 5'968 (16.9) | 29'182 (22.2) | 9'480 (19.4) | 37'363 (34.5) |
| Managed care (%) | 13'056 (55.8) | 132'386 (55.6) | 20'024 (56.6) | 71'300 (54.3) | 26'450 (54.1) | 59'763 (55.1) |
| Suppl. hospital insurance (%) | 5'932 (25.3) | 50'407 (21.2) | 9'871 (27.9) | 32'447 (24.7) | 12'374 (25.3) | 20'492 (18.9) |
| Language region |  |  |  |  |  |  |
| German (%) | 17'650 (75.4) | 184'079 (77.3) | 21'616 (61.1) | 105'467 (80.3) | 35'101 (71.8) | 87'167 (80.4) |
| French (%) | 3'567 (15.2) | 37'412 (15.7) | 9'360 (26.4) | 17'439 (13.3) | 8'756 (17.9) | 14'635 (13.5) |
| Italian (%) | 2'195 (9.4) | 16'779 (7.0) | 4'412 (12.5) | 8'381 (6.4) | 5'002 (10.2) | 6'608 (6.1) |
| Urban region (%) | 18'361 (78.4) | 181'330 (76.1) | 27'740 (78.4) | 101'169 (77.1) | 38'004 (77.8) | 80'953 (74.7) |
| Major related surgery/ disease (%) | 274 (1.2) | 716 (0.3) | 2'073 (5.9) | 1'207 (0.9) | 2'223 (4.5) | 918 (0.8) |
| Chronic conditions  (mean, sd) | 1.8 (1.83) | 1.4 (1.69) | 1.8 (1.87) | 1.6 (1.84) | 2.0 (1.77) | 1.3 (1.65) |
| Cantonal program (%) | 1'273 (5.4) | 13'261 (5.6) | 23'181 (65.5) | 61'371 (46.7) | 0 (0) | 0 (0) |

^a^ FOBT = fecal occult blood testing

^b^ PSA = prostate-specific antigen
