# Supplementary figures and images for: Temporal trends and regional disparities in cancer screening utilization: an observational Swiss claims-based study
Source: BMC Public Health. 2021 Jan 5;21:23. doi: 10.1186/s12889-020-10079-8 (PMC7786957; doi:10.1186/s12889-020-10079-8)

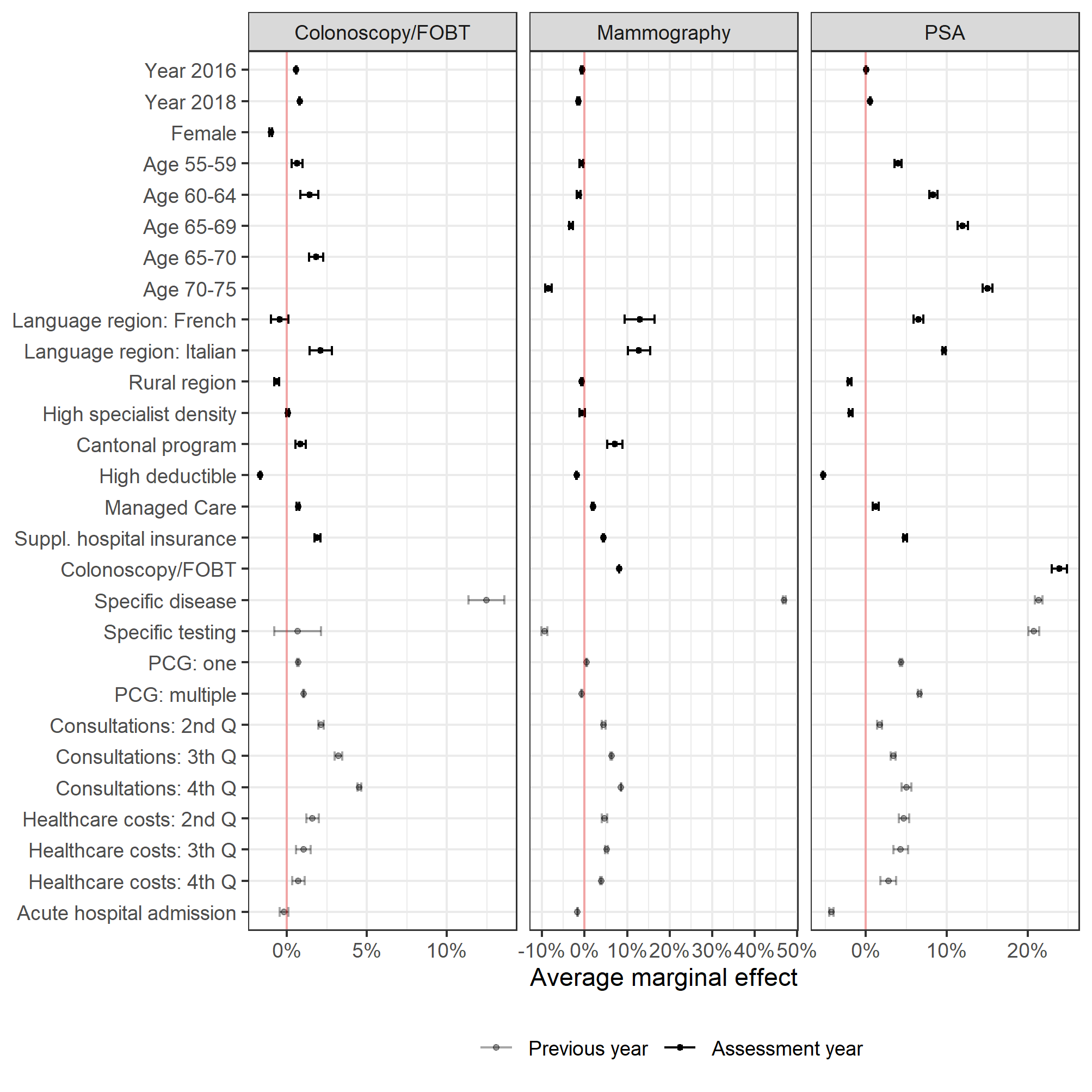

Supplement: Supplementary file 3 — Additional file 3. Estimates with clustered covariance of the average marginal effects on colonoscopy/FOBT, mammography and PSA testing utilization. [file 12889_2020_10079_MOESM3_ESM.tiff]

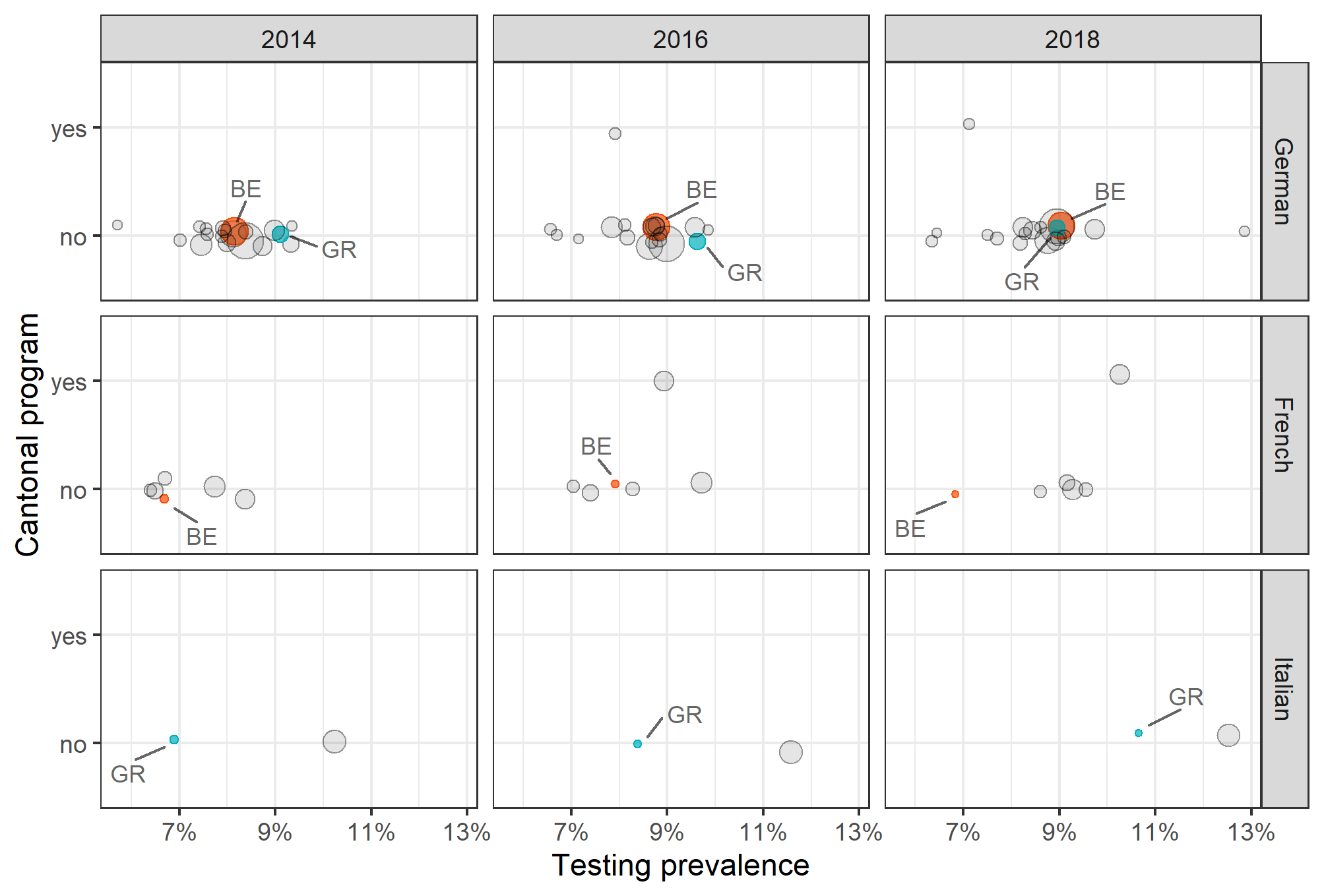

Supplement: Supplementary file 4 — Additional file 4. Age-standardized proportions of colonoscopy/FOBT utilization per canton, by language region and cantonal screening program. The size of the bubbles corresponds to the population size of the canton; BE = canton of Bern; GR = canton of Grisons. The age-standardized proportion of persons with colonoscopy/FOBT mainly differed between the German- and the Italian-speaking regions. By the end of 2015, only two cantons offered a specific program, of which one canton (Uri) is minor. In the canton of Vaud, the colonoscopy/FOBT utilization increased between 2016 and 2018. [file 12889_2020_10079_MOESM4_ESM.tiff]

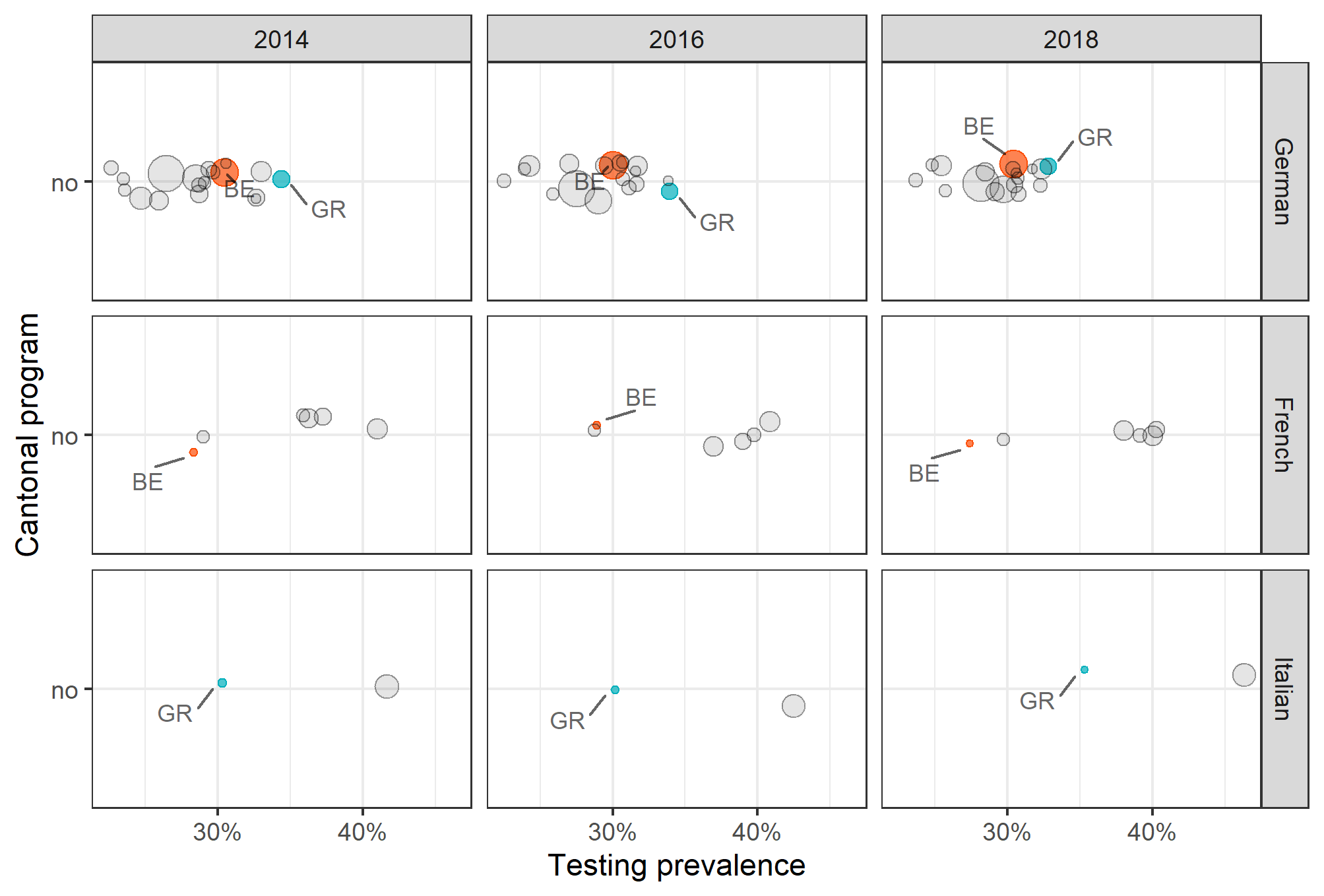

Supplement: Supplementary file 5 — Additional file 5. Age-standardized proportions of PSA testing utilization per canton, divided by language region. The size of the bubbles corresponds to the population size of the canton; BE = canton of Bern; GR = canton of Grisons. Regarding prostate cancer screening, where no cantonal programs exist, PSA testing utilization seems higher in the French- and Italian-speaking compared to the German-speaking regions. Then again, the testing proportions in the French-speaking regions of BE and the Italian-speaking region of GR are more comparable to the German-speaking region of the respective canton. [file 12889_2020_10079_MOESM5_ESM.tiff]
